# Epidemiology of Adult T-Cell Leukaemia/Lymphoma in South Africa over a 10-Year Period

**DOI:** 10.1155/2022/2058280

**Published:** 2022-08-31

**Authors:** Erica-Mari Nell, Ibtisam Abdullah, Carla Griesel, Nadhiya Subramony, Louis Almero du Pisani, Zivanai Cuthbert Chapanduka

**Affiliations:** ^1^Division of Haematological Pathology, Department of Pathology, Faculty of Medicine and Health Sciences, Stellenbosch University, Cape Town, South Africa; ^2^National Health Laboratory Service, Tygerberg Hospital, Cape Town, South Africa; ^3^Division of Haematology, Department of Pathology, Northland District Health Board, Northland, New Zealand; ^4^Ampath Laboratories, Cape Town, South Africa; ^5^Lancet Laboratories, Johannesburg, South Africa; ^6^PathCare Laboratories, Cape Town, South Africa

## Abstract

**Introduction:**

Adult T-cell leukaemia/lymphoma (ATLL) is a rare and aggressive malignancy of mature T-cells. Limited epidemiological studies have shown that there is substantial variation in age at diagnosis and subtype distribution between different geographical regions. This is the first epidemiological study of ATLL in South Africa.

**Methods:**

A national epidemiological study of ATLL in South Africa was performed. All new cases of ATLL from 2009 to 2019 were identified by laboratory database search in public and private health care sectors. Demographic and diagnostic data were obtained, and the cases were subtyped according to the Shimoyama classification.

**Results:**

There were 31 patients with ATLL over the 10-year period, with an incidence of 0.06 per 100000 population. The male to female ratio was 1 : 1 and the median age at diagnosis was 37 years. Acute ATLL was the most commonly seen subtype in South Africa.

**Conclusion:**

In this, the first epidemiological study of ATLL in South Africa, we demonstrate that ATLL is a rare disease, that acute ATLL is the most commonly diagnosed subtype, and that ATLL is likely under diagnosed. Patients present at a considerably younger age than the reported age in other nations.

## 1. Introduction

Adult T-cell leukaemia/lymphoma (ATLL) is a rare malignancy of mature CD4+ T-cells. It is subclassified according to the Shimoyama classification into 4 subtypes [1]. The clinically aggressive variants are the acute and lymphomatous subtypes, while the clinically indolent variants are the chronic and smouldering subtypes [1].

Japan has the highest prevalence of ATLL in the world, and thus, the epidemiology of HTLV-1 and ATLL in Japan is well described [2, 3]. The acute subtype is the most common subtype of ATLL in Japan, seen in 49.5% to 56.1% of patients (*n* = 922, collected over a 2-year period, and *n* = 1594, collected over 9-year period, respectively) [4, 5]. In contrast, the lymphomatous subtype is the most common subtype in the USA (49.2%, *n* = 195, collected over a 30-year period) and Latin America (50.2%, *n* = 253, collected over a 25-year period) [6, 7]. In Japan, the median age at diagnosis is 61 to 70 years across the different subtypes of ATLL [4, 5]. In contrast, in Latin America and the USA, the median age at diagnosis is more than a decade younger, at 52 to 57 years across ATLL subtypes, with one study in Brazil showing a median age as low as 44 years (*n* = 369, collected over a 31-year period) [6–8].

Human T-lymphotropic virus type 1 (HTLV-1) is aetiologically linked to ATLL, and as such, the geographic distribution of ATLL corresponds with that of HTLV-1 endemic areas [9]. The epidemiology of ATLL is poorly described in South Africa despite South Africa being an HTLV-1 endemic area, with an HTLV-1 seroprevalence of 0.062% [10]. The difference in the median age at diagnosis between different geographic regions suggests that epidemiological data is not generalisable across different geographical regions. This study was performed to describe the epidemiology of ATLL in South Africa.

## 2. Material and Methods

### 2.1. Study Setting and Design

A national retrospective descriptive study was performed over a 10-year period. All new ATLL cases that were diagnosed in South Africa from August 2009 to August 2019 were identified for inclusion in our study. Data was collected in the public health care sector as well as the private health care sector in order to perform a national epidemiological study on ATLL.

Within South Africa, there is no standard investigation algorithm for diagnosis of T-cell lymphomas and each hospital follows their local protocol.

### 2.2. Data Collection

Public health care sector data was retrieved from the National Health Laboratory Service (NHLS) central data warehouse (CDW), which houses patient results from all the provinces of South Africa. The NHLS provides diagnostic laboratory services to about 80% of the South African population [11]. All laboratory information system (LIS) entries containing the phases/keywords “ATLL,” “ATL,” “adult T-cell leukemia,” “adult T-cell lymphoma,” “adult T-cell leukaemia/lymphoma,” or part thereof were extracted by the NHLS CDW. In all episodes where ATLL was the preferred diagnosis or a part of the differential diagnosis, the cases were manually reviewed on the NHLS LIS to confirm the diagnosis of ATLL and collect supporting diagnostic data and demographic information for each case. The histology, peripheral blood morphology, immunophenotype, clinical presentation, and HTLV-1 status were reviewed for diagnosis of ATLL. Additionally, the lymphocyte count, abnormal lymphocyte percentage, LDH, and calcium levels were used to classify patient cases according to the Shimoyama criteria [1]. HTLV-1 testing was performed on serum by polymerase chain reaction (PCR).

For the private health care data, coinvestigators from Ampath, Lancet, and PathCare private laboratories collected national data on ATLL over the study period at their respective laboratories via their respective databases.

### 2.3. Data Analysis

All confirmed cases of ATLL were transferred to a computer spreadsheet (Microsoft Excel, Microsoft Corp., Redmond, WA). Frequencies were calculated for categorical data. Median and interquartile range were calculated for age. Incidence was calculated using the 2009 South African population and a 95% confidence interval was calculated as previously published [12].

### 2.4. Ethical Considerations

This study was approved by the Stellenbosch University Human Research Ethics Committee review board (N19/09/115), and all research was performed in accordance with relevant regulations with anonymised data. The requirement for informed consent was waived by the ethics committee due to the retrospective nature of the study.

## 3. Results

**Figure 1:**
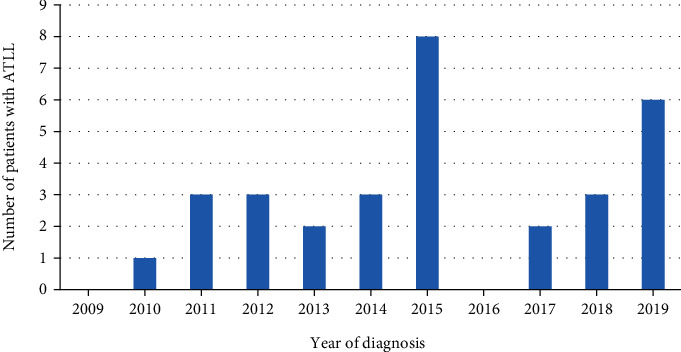
Number of patients diagnosed with adult T-cell leukaemia/lymphoma (ATLL) per year.

There were 31 patients diagnosed with ATLL over the 10-year period (Figure 1), an average of 3 per year. This represents an incidence of 0.06 per 100000 population (95% CI 0.04–0.09 per 100000). Two of these were seen in the private health care sector and the remaining 29 patients were seen in the public health care sector.

**Figure 2:**
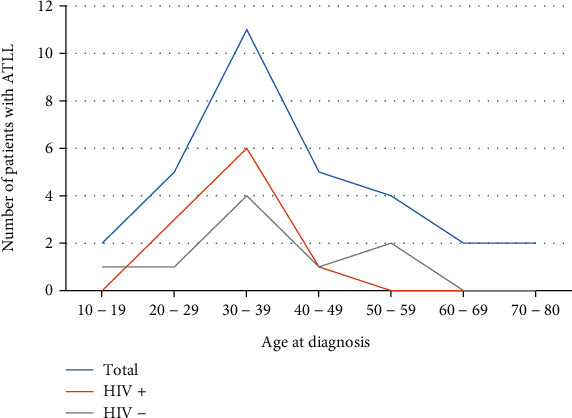
Distribution of age at diagnosis of ATLL for the total number of patients, as well as those that are known to be HIV positive or HIV negative. ATLL: adult T-cell leukaemia/lymphoma; HIV: human immunodeficiency virus.

The median age at diagnosis was 37 years (interquartile range 30–49; range 17–80). Ten patients were HIV positive, 9 patients were HIV negative, and the HIV status of 12 patients was unknown. The age distribution was similar in patients that were HIV positive and HIV negative (Figure 2). The male to female ratio was 1 : 1 (15 males and 16 females). Unfortunately, ethnicity data is not captured routinely onto the LIS and there is insufficient data to comment on ATLL distribution according to ethnicity.

Lymphocytes with “flower-like” nuclear morphology were observed on peripheral blood in 53% of patients (16/30). Flow cytometry was performed in 23 of the patients with ATLL. The flow cytometry showed the typical immunophenotype (positive for CD3, CD4, CD2, CD5, and CD25 and negative for CD7 and CD8) in 74% (17/23). In 5 cases, CD7 was expressed. Three cases of ATLL expressed both CD4 and CD8. Two cases did not express CD25.

Acute ATLL was the most common subtype seen (26/31; 84%). There were two patients with smouldering ATLL, two patients with chronic ATLL, and one with lymphomatous ATLL. In the patients with acute ATLL, all but two had bone marrow involvement and the patient with lymphomatous ATLL also had bone marrow involvement. The patients with chronic and smouldering ATLL did not have a bone marrow examination as part of their diagnostic work up. A skin biopsy was performed in seven patients, six of these confirming cutaneous involvement by ATLL. Lymph node biopsies were performed in three patients confirming lymph node involvement by ATLL. HTLV-1 PCR was requested in 20 of the 31 patients and was positive in all 20 cases.

## 4. Discussion

This is the first national epidemiological study of ATLL in South Africa. The incidence was 0.06 per 100000 population over the 10-year period, the majority of these being the acute subtype.

The lifetime risk of developing ATLL in a person with HTLV-1 infection is 3–5%, as calculated by studies in Japan where HTLV-1 and ATLL are prevalent [5, 13]. The number of patients with ATLL that we should be seeing in South Africa, based on this lifetime risk and the prevalence of HTLV-1 in South Africa, can therefore be calculated [8, 14]. Over the study period, the population of South Africa was 50.48 million (2009) to 58.76 million (2019) [15]. The prevalence of HTLV-1 in South Africa is 0.062%, and therefore, we estimate that 31000 to 36000 persons in South Africa have HTLV-1 infection [10]. Taking into account the South African life expectancy of 64 years [15], this means that we should see 15 to 24 cases of ATLL in 2009 and 17 to 28 cases of ATLL in 2019 in South Africa.

However, far fewer cases of ATLL were seen in this epidemiological study, averaging 3 per year over the 10-year period. The discordantly low incidence is likely due to under diagnosis of ATLL. The reasons for this are likely related to challenges of diagnosis within a limited resource setting. In patients with aggressive ATLL subtypes, the diagnosis may not be made as patients may demise before definitive investigation can be performed. Furthermore, patients with the non-acute forms of ATLL may not be appropriately referred from primary health care facilities for diagnosis. In addition, it was noted that even in the patients who had suggestive histology/immunophenotype for ATLL, only 20 of the 31 patients had testing for HTLV-1. There may have been other T-cell lymphomas that were misclassified because HTLV-1 testing was not performed. This highlights the need for ongoing education of health professionals regarding proper investigation and referral of patients with suspected lymphoma and routine testing of all patients with a T-cell lymphoma for HTLV-1, so that ATLL is not missed. The much lower rate of ATLL diagnoses compared to HTLV-1 infection seroprevalence is also noted in epidemiological studies in Brazil and the Netherlands, and this was similarly attributed to under diagnosis [8, 14]. This highlight the need for standardisation of diagnostic methods in patients with suspected non-Hodgkin lymphoma and especially T-cell lymphoma across international borders.

Another possibility contributing to the discordantly low incidence of ATLL may be that the life-time risk of developing ATLL in individuals with HTLV-1 infection that was calculated in Japanese studies is not translatable to our population. Development of ATLL is associated with mother-to-child transmission of HTLV-1, rather than acquiring HTLV-1 later in life [16]. From the South African HTLV-1 prevalence study, it is evident that HTLV-1 seroprevalence is higher in older age groups compared to younger age groups [10]. This may indicate that HTLV-1 infection is acquired later in life, and thus, the life-time risk of ATLL in South Africans may be lower than the life-time risk calculated in studies conducted in Japan. Another consideration is that the median age of ATLL in Japan is 68 years, which is similar to South Africa's life expectancy of 64 years [5, 15]. This may indicate that individuals in South Africa are not living long enough to develop ATLL.

In this study, acute ATLL was the most common subtype of ATLL accounting for 84% of the ATLL cases. There was only one patient with lymphomatous ATLL in South Africa over the 10-year period, despite lymphomatous ATLL being relatively common in other geographic regions [4–7]. While in Japan, acute ATLL is also most commonly seen, accounting for approximately half of their ATLL cases, lymphomatous ATLL is the second most common seen in approximately a quarter of ATLL cases [4, 5]. Additionally, in the USA and Latin America, lymphomatous ATLL is the most common subtype, accounting for approximately half of their cases [5, 6]. Considering this international context, it is likely that lymphomatous ATLL is underdiagnosed in South Africa. Given the high tuberculosis burden in South Africa, lymphadenopathy is often attributed to and empirically treated as tuberculosis [17] and patients may not be appropriately referred from primary health care facilities for further investigations, including radiological imaging or biopsy of lymphadenopathy, required for diagnosis of lymphoma. This highlights the need for ongoing education of health professionals to seek definitive diagnosis of lymphadenopathy so that lymphoma is not incorrectly treated as tuberculosis.

In this study, ATLL was seen equally in male and female patients, as is seen across many geographical regions [5, 7, 8, 18]. The median age at diagnosis of ATLL is 37 years in South Africa, much lower than the 6^th^ decade of life seen in Japan, the 5^th^ decade in the USA and Latin America, and still lower than the median age of 44 years seen in a Brazilian study [4–8]. Given that South Africa has a high HIV prevalence, we hypothesised that coinfection of HTLV-1 with HIV may have a role in expediting the pathogenesis of ATLL; however, the age distributions between HIV-positive and HIV-negative persons were similar. The small numbers involved in this study are noteworthy. Other environmental factors or social factors may be implicated in the lower age at diagnosis in South Africa. Awareness that the majority of patients with ATLL in South Africa were in the 3^rd^ decade of life is important to ensure that a diagnosis of ATLL is not missed or delayed based on unlikely age.

This study is the first South African national study to investigate the incidence of ATLL in the South African population. The limitations of this study include the limited access to information regarding ethnicity, social characteristics, family history of ATLL or HTLV-1 carriers, and exposure to other infections and toxins which may have an impact on the age of ATLL diagnosis. Availability of this data may aid identification of risk factors of ATLL in our population.

In conclusion, this epidemiological study of ATLL in South Africa showed a national incidence of 0.06 per 100000 population over the 10-year period. Acute ATLL is the most commonly diagnosed subtype in South Africa, and ATLL, especially lymphomatous ATLL, is likely under diagnosed. Women and men are equally affected, and the median age at diagnosis is 37 years, much lower than other parts of the world. This epidemiological information is important to facilitate prompt diagnosis of this high mortality disease. The factors that expedite the evolution of ATLL in our population are not fully established. Further research is required to understand the precipitating factors contributing to the oncogenic process.

## Data Availability

All data is available in the manuscript.
